# Functional Impact of Risk Gene Variants on the Autoimmune Responses in Type 1 Diabetes

**DOI:** 10.3389/fimmu.2022.886736

**Published:** 2022-05-04

**Authors:** Chelsea Gootjes, Jaap Jan Zwaginga, Bart O. Roep, Tatjana Nikolic

**Affiliations:** Laboratory of Immunomodulation and Regenerative Cell Therapy, Department of Internal Medicine, Leiden University Medical Center, Leiden, Netherlands

**Keywords:** type 1 diabetes, risk gene variants, immunoregulation, Tregs, tolerogenic dendritic cells, vitamin D

## Abstract

Type 1 diabetes (T1D) is an autoimmune disease that develops in the interplay between genetic and environmental factors. A majority of individuals who develop T1D have a HLA make up, that accounts for 50% of the genetic risk of disease. Besides these HLA haplotypes and the insulin region that importantly contribute to the heritable component, genome-wide association studies have identified many polymorphisms in over 60 non-HLA gene regions that also contribute to T1D susceptibility.

Combining the risk genes in a score (T1D-GRS), significantly improved the prediction of disease progression in autoantibody positive individuals. Many of these minor-risk SNPs are associated with immune genes but how they influence the gene and protein expression and whether they cause functional changes on a cellular level remains a subject of investigation. A positive correlation between the genetic risk and the intensity of the peripheral autoimmune response was demonstrated both for HLA and non-HLA genetic risk variants. We also observed epigenetic and genetic modulation of several of these T1D susceptibility genes in dendritic cells (DCs) treated with vitamin D3 and dexamethasone to acquire tolerogenic properties as compared to immune activating DCs (mDC) illustrating the interaction between genes and environment that collectively determines risk for T1D. A notion that targeting such genes for therapeutic modulation could be compatible with correction of the impaired immune response, inspired us to review the current knowledge on the immune-related minor risk genes, their expression and function in immune cells, and how they may contribute to activation of autoreactive T cells, Treg function or β-cell apoptosis, thus contributing to development of the autoimmune disease.

## Introduction

For several decades the incidence of Type 1 diabetes (T1D) has been increasing worldwide (1). This disease is characterized by the infiltration of immune cells in the islets of Langerhans (2, 3) ultimately leading to the loss of insulin producing β-cells with insulin replacement as the only available option to prevent fatal outcomes in all patients. Curative treatments are lacking for several reasons, one being that the events in humans leading to a selective β-cell dysfunction and loss is hard to detect. Although the analyses of fresh and cryopreserved tissues in the past decade, enabled by the nPOD initiative (www.JDRFnPOD.org), have significantly contributed to our understanding of the important local players in the process (2–5), many questions remain. So far, adaptive immune cells are indisputably involved in the β-cell destruction by their tissue specificity. The clinically approved therapies modulate immunity in general, the more preferable antigen-specific immune therapies show promising results but are not ready for general clinical application (6).

While autoimmune T1D is not completely inherited and environmental factors show a significant contribution to the pathogenesis (7), certain genetic polymorphisms do critically increase the predisposition for T1D (8). Polymorphisms in HLA and insulin (INS) regions were first described and contribute strongly to the disease risk (9, 10). Later, genome-wide association studies (GWAS) have identified many additional SNPs in so called non-HLA risk genes, which show a small but clear individual contribution to the increased risk for T1D (11). When included in a cumulative score (T1D-GRS), it significantly improved the capacity to discriminate T1D from T2D or healthy subjects, and to discriminate monogenic from autoimmune T1D (12–14). The exact functional contribution of many of these SNPs to the T1D-GRS remains to be characterized. We have observed a positive correlation between the non-HLA genetic risk, in addition to, but independently of HLA, and the intensity of the peripheral autoimmune response in T1D patients (15). Indeed, many of the associated T1D risk genes are controlled by lymphoid enhancers or involved in immune networks (11, 16). Our studies focusing on the differential transcriptome of tolerogenic (tolDC) versus inflammatory dendritic cells (mDCs) showed that a tolerogenic modulation of monocytes by 1,25(OH)_2_ vitamin D3 (VitD3) induced a stable change in the expression of sets of these non-HLA risk genes (17, 18), inspiring a hypothesis that quantitative and/or qualitative effects of the SNPs on the related gene products may reflect in a change of the immune regulatory vs. an immune activating balance. Here, we aim to review the knowledge of functional consequences of T1D risk SNPs on the regulation, expression and function of linked risk genes and further contemplate how this may impact the functionality of the effector vs. regulatory T cells, changing the balance between immune activation and suppression in the pancreas that is critical to attenuate chronic inflammation and an uncontrolled damage of insulin producing β-cells.

## Immunopathogenesis of T1D, How Much Do We Actually Know?

The exact order of immune events that cause human T1D has not been established. Hurdles such as that circulating blood cells poorly represent what is going on in the human pancreas, as well as the inability to directly analyze the target organ have significantly delayed our understanding of this autoimmune disease. Literature supports different scenarios describing the initiating events, involving an altered thymic selection of T cells that recognize β-cell antigens, viral infections that mark β-cells as the targets for destruction, enhanced expression of neo-antigens by β-cells due to cell stress, or an increased sensitivity of β-cells to inflammation (19–21). In all cases, β-cells seem critically involved in the process while the (auto)immune system is essential to execute the destructive insult resulting eventually in disease (22). The proposed initiating events are not mutually exclusive and likely cause the exposure of β-cell (neo-)antigens, which are taken-up by antigen presenting cells and presented to T cells in the context of high-risk human leukocyte antigen (HLA) molecules. The ‘first hit’ could occur when such presentation of β-cell antigens activates a destructive autoimmune response that may inflict some damage in the pancreatic islets but the disease is delayed as long as the immune regulation keeps the autoimmunity in check. The ‘second hit’ may occur when a regulatory checkpoint is bypassed such as upon an anti-cancer stimulating immunotherapy by check point inhibition or when the stressed (or infected) islets of Langerhans start releasing cytokines and chemokines, attracting immune cells where they target and eliminate functional β-cells to the point of no return.

T1D patients donating tissues for research helped to collect a significant evidence that T1D pathogenesis can follow different individual scenarios but also that mechanisms described in animal models are not all or not just as strongly present in the human immune system. For example, the infiltration of immune cells around the islet of Langerhans, designated as insulitis, in which activated CD4+ T cells control macrophages to induce killing of the β-cells by cytokines and reactive oxygen species, is clearly evident in mice (23) but not evident in human pancreas (24, 25). Cytotoxic CD8+ T cells are essential for the ultimate destruction of β-cells, while their antigen specificity varies between patients (4, 26). Hypothetically, techniques that discriminate relevant antigens and cells in the target tissue, allowing to separate primary immune aggressors from those only guilty by association will help solve this puzzle. Such bystanders may be the autoantibodies, which role in the immunopathology of human T1D is proved dispensable although they represent a good biomarker of an ongoing and in time often increasingly complex autoimmunity (27, 28). These antibodies can be found months to years before the clinical symptoms (29), help an early diagnosis of the disease and may prove valuable to identify individuals that will benefit from new curative treatments.

Time will tell whether the knowledge on the specificity of T1D autoantibodies to insulin (INS), 65 kDa glutamic acid decarboxylase (GAD65), insulinoma-associated protein2 (IA2) and zinc transporter 8(Znt8) (30) has helped or derailed the investigation of the β-cell specific targets of T cell autoimmunity (26, 31). More recent findings point to alternative transcripts and (neo-)antigens created by stressed or damaged β-cells, which are normally invisible to the immune system, as more likely to drive the T-cell mediated pathogenic destruction (21, 32–37). The contribution of the originally described antigens could be different, namely to secure immune regulation through a negative selection of high-affinity autoreactive T cells (38), or to establish peripheral tolerance through low-affinity self-peptide recognizing regulatory T cells (Tregs) (39, 40). The existence of autoantibodies may hence be a sign of a regulation ‘gone wrong’ as a consequence of a genetically imprinted or environmentally caused impaired T cell selection, effector activation or reduced Treg function, such as demonstrated in T1D patients (41, 42).

## The Impact of Major T1D Risk Genes on Immune Cells

Certain genetic polymorphisms associate with a higher risk to develop an autoimmune disease, which is most often expressed as an odds ratio (OR) that measures the strength of association between carrying a gene variant X (exposure) and development of T1D (outcome) (43). Specific HLA haplotypes and SNPs in the insulin gene (INS) strongly increase the odds to develop T1D and are hence designated as major susceptibility genes (44, 45). The HLA region was first associated with the risk of developing T1D, which is in line with a critical role of HLA in shaping the adaptive immunity (46). In the population of Caucasian origin, more than 90% of patients that develop T1D before puberty are carriers of one or both HLA haplotypes, namely HLA-DR3/DQ2 (DRB1*0301-DQA1*0501-DQB1*0201) or HLA-DR4/DQ8 (DRB1*0401-DQA1*0301- DQB1*03020 (47). In fact, heterozygotes carriers of both DRB1*03 and DRB1*04 carry up to 40 times higher risk to develop T1D than individuals with other HLA genotypes (48, 49). This synergic effect is likely caused by the formation of highly susceptible *trans*-encoded HLA-DQ (α1, β1) heterodimer molecules (48, 50), which efficiently bind and present β-cell derived peptides, increasing the number of different peptides that could trigger a pathogenic CD4+ T cell responses (51). Furthermore, the risk variant specific epigenetic modulation of the HLA expression could contribute to the disease pathogenesis (52).

A stable HLA molecule on the cell surface, however, does not exist without a peptide. Hence the contribution of HLA should be considered in combination with antigens/peptides that they present. The so far well-established β-cell antigens that are targeted by both B and T cell responses are INS, GAD65, IA2 and Znt8 but the list of target antigens is increasing (34, 35, 53). Of the β-cell proteins targeted as autoantigens, only SNPs in the INS gene are associated with an increased risk for T1D. The increased risk was first attributed to the polymorphism in variable number of tandem repeats (VNTR) in the insulin promotor (54, 55), determining the differential insulin expression between thymus and islets and leading to a faulty selection of the autoreactive T cells in thymus. While this may explain a part of the association, alternatives have been also explored, one being that other SNPs in the 3’ UTR of the INS gene (rs3842752 (56) and rs3842753 (57)) actually functionally contributes to the increased risk. Namely, these SNPs are expressed when an alternative translation start in the INS mRNA is used, creating a new protein sequence called INS-DRIP. Interestingly, a few T1D patients carrying the protective allele (C-H) demonstrated no autoreactivity to INS-DRIP unlike the carriers of the susceptible (R-P) version (36). Which insulin-related SNP is causal and whether the increased risk is a consequence of the expression of 3’SNPs in INS-DRIP or it reflects the 5’ INS promotor polymorphism remains unresolved, given the strong linkage disequilibrium between the 5’and 3’ regions of INS, and the exact underlying mechanism is currently under investigation.

Despite the critical role of CD8+ T cells, the contribution of HLA class I molecules to the disease propensity is less obvious and affected by the high linkage disequilibrium between HLA class I and II genes. For instance, 50-70% of T1D patients carry HLA-A2 (0201), which turns this HLA class I allele as the most frequent amongst patients; yet, this variant is also present in 30-40% of the general population, affecting the statistical significance. HLA-B*39 has been identified as single HLA class I allele standing out in its association with T1D, but this variant is relatively rare (58). In our view, this indicates a more important role of HLA class II and antigen presentation in establishing and control of the immune regulation than in the actual β-cell destruction.

## Minor T1D Risk SNPs With a Functional Impact on Immune Cells

**Figure 1:**
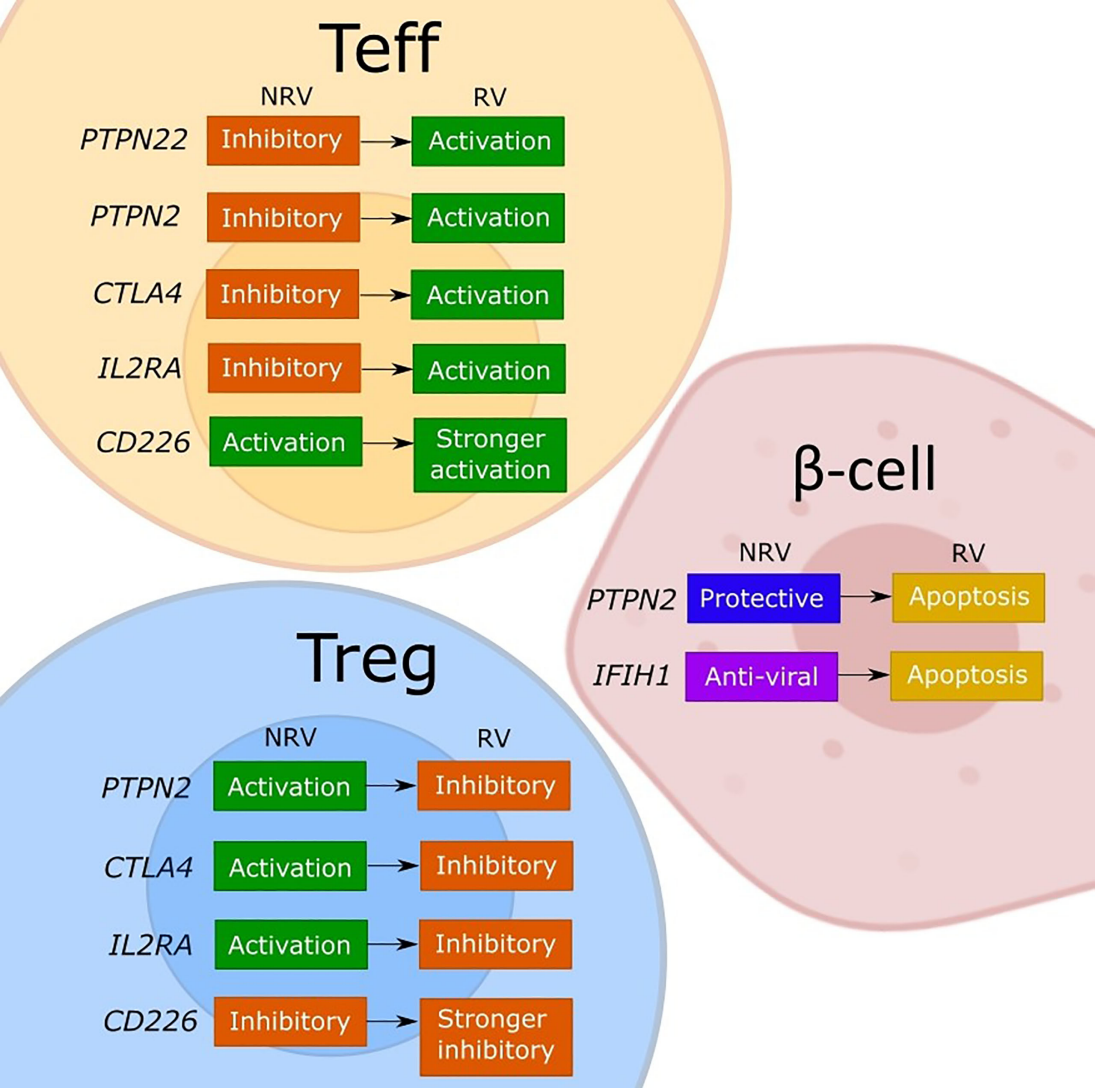
Model of the discussed effects of T1D risk variants on cellular functions. The figure depicts our interpretation of the consequences for effector T cell (Teff), regulatory T cell (Tregs), and β-cells of the described or assumed change in the gene function caused by a T1D risk variant (RV) as compared to the non-risk variant (NRV) SNP as discussed in the manuscript. While the LYP protein normally controls the effector T cells by a downstream signaling inhibition, the risk variant (rs2476601) induces a change in PTPN22 that promotes Teff responses. The functional effects of PTPN22 remain unclear. The PTPN2 protein plays an anti-apoptotic role in β-cells and controls T cells *via* IL-2, which may favor Tregs due to a strong sensitivity to IL-2. Indirectly, a good activity of Tregs keeps the effector T cells under control. The *PTPN2* risk variant (rs1893217) causes a decrease in *PTPN2* expression and contributes to the sensitivity of β-cells to immune- or virus-mediated apoptosis. The risk variant also reduces IL-2 receptor signaling, which decreases FOXP3+ Tregs in T1D patients, and thus dysregulating Treg function. The *PTPN2* deficiency (mimicking the rs1893217 variant) results in increased Teff proliferation. The MDA5 (encoded by *IFIH1*) normally functions to activate stress- and anti-viral response, and by increasing the activity of MDA5, the risk variant (rs1990760) increases the basal IFN-I production leading to β-cell apoptosis. CTLA-4 functions normally to promote Treg function and inhibit Teff activation. The risk variant for *CTLA4* (rs231775) results in decreased expression of CTLA-4 on T cells, releasing the control of a Teff cell activation and reducing the suppressive Treg potency. The *IL2RA* risk variants impair the expression of CD25 and thus the IL-2 response and with the associated lower FOXP3 expression impacts primarily Tregs and their suppressive function. The resulting reduced Treg potency will indirectly release the control on Teff promoting the activation. The CD226 is an activating T cell molecule that promotes the inflammatory activity of Teff and reduces the suppression of Tregs. The *CD226* risk variant (rs763361) results in an isoform of CD226 with increased activity, which further increases Teff and CD226+ Tregs, thereby further reducing the overall suppressive capacity of Tregs.

For many risk genes variants, there is still insufficient understanding of whether and how they functionally impact the initiation and progression of the autoimmune process causing T1D. The functional outcomes of the coding T1D risk variants have been reviewed recently (59), and a fine mapping of the 10 known susceptibility regions combined with functional analyses provided further insight in potentially causal missense and non-coding SNP variants (60). Many of these risk genes were differentially expressed in dendritic cells upon tolerogenic modulation (17, 18). Hence, we here consider the functional roles in immune regulation of the minor T1D risk genes as such or when influenced by the SNP. We mainly focus on the genes for which functional data on human cells are available to allow a discussion on the consequences of the causal SNPs for the autoreactive T cell activation, Treg function or β-cell apoptosis that may support the autoimmune disease (**Table 1** and **Figure 1**).

**Table 1:** Risk gene variants associated with T1D (discussed in this review). For each gene variant the variant ID, risk allele frequency and odd ratio are presented.

Gene	Variant ID (RSID)	OriginalPub.*		Frequency**	Odds Ratio	Assoc. p-value	Publication***
HLA class II	DRB1* 04:05-DQA1*03:02-DQB1*03:02				11.370	4.000 x 10^-5	Erlich H et al., 2008 (61)
	DRB1* 04:01-DQA1*03:01-DQB1*03:02				8.390	6.000 x 10^-36	Erlich H et al., 2008 (61)
	DRB1* 03:01-DQA1*05:01-DQB1*02:01				3.640	2.000 x 10^-22	Erlich H et al., 2008 (61)
	DRB1* 04:02-DQA1*03:01-DQB1*03:02				3.630	3.000 x 10^-4	Erlich H et al., 2008 (61)
*INS*	rs689	(62)	A –> T	T: 68%	2.256	2.161 x 10^-135	Inshaw JRJ et al., 2021 (63)
	rs3842752	(56)	G –> A	A: 20%	0.600	2.310 x 10^-14	Reddy et al., 2011 (56)
	rs3842753	(57)	T –> G	G:70%	0.580	2.180 × 10-32	Howson et al., 2009 (57)
*PTPN22*	rs2476601	(64)	A –> T	T: 9%	1.890	1.000 x 10^-100	Onengut-Gumuscu S et al., 2015 (11)
*PTPN2*	rs1893217	(65)	A –> G	G: 15%	1.210	1.200 x 10^-15	Onengut-Gumuscu S et al., 2015 (11)
*IFIH1*	rs2111485	(66)	A –> G	G: 57%	1.171	1.892 x 10^-10	Forgetta V et al., 2020 (67)
	rs1990760	(68)	C –> T	T: 57%	1.180	2.000 x 10^-11	Todd JA et al., 2007 (69)
	rs3747517	(66)	T –> C	C: 71%	1.700	6.000 x 10^-4	Liu S el al., 2009 (70)
	rs13422767	(70)	G –> A	A: 15%	1.799	1.000 x 10^-4	Zurawek M et al., 2015 (71)
*CTLA4*	rs231775	(72)	A –> G	G: 37%	2.000	1.000 x 10^-2	Goralczyk A et al., 2018 (73)
	rs5742909	(74)	C –> T	T: 8%	1.500	2.000 x 10^-2	Chen S et al., 2019 (75)
	rs3087243	(69)	G –> A	A: 44%	0.840	7.400 x 10^-21	Onengut-Gumuscu S et al., 2015 (11)
*IL2RA*	rs11594656	(76)	T –> A	T: 77%	1.220	1.920 x 10^-28	Lowe CE et al., 2007 (76)
	rs2104286	(77)	T –> C	C: 24%	0.880	2.100 x 10^-2	Espino-Paisan L et al., 2011 (78)
	rs12722495	(79)	T –> C	C: 8%	0.620	1.740 x 10^-30	Smyth DJ et al., 2008 (79)
	rs61839660	(76)	C –> T	T: 9%	0.620	2.800 × 10^−39	Onengut-Gumuscu S et al., 2015 (11)
*CD226*	rs763361	(69)	C –> T	T: 48%	1.120	1.000 x 10^-9	Plagnol V et al., 2011 (80)

Data in this table has been collected using the database on https://platform.opentargets.org for type 1 diabetes mellitus. Genetic associations were selected as data type and Immune system as pathway types. Per gene variant the odds ratio is derived from the study listed in the OT Genetics Portal. *The original paper reporting the association between the risk variant and T1D. **Frequency of a risk allele in the world. ***Publications have been cited which reported the OR and p-value in the table.

## PTPN22

Protein tyrosine phosphatase, non-receptor type 22 (*PTPN22)* encodes protein Lymphoid-tyrosine phosphatase (LYP) (81). The *PTPN22* allele C1858T has a single amino acid substitution R620W (arginine to tryptophan; rs2476601, OR= 1.890), and has been associated with T1D, Hashimoto’s thyroiditis, rheumatoid arthritis (RA), systemic lupus erythematosus (SLE), vitiligo and Graves disease (82). The linkage with several autoimmune diseases and the observation that individuals with this variant are protected from pulmonary tuberculosis or cancer (83, 84), suggests a role in promoting effector responses at cost of immune regulation (85–88). LYP protein inhibits T cell and B cell activation by dephosphorylation of tyrosine residues in Src family kinases. The interaction between C-terminal Src kinase (CSK) and the P1 motifs on LYP are important to regulate the inhibitory activity of LYP. Next to lymphocytes, LYP plays a role in the control of activation and migration of innate myeloid cells (monocytes, macrophages, DCs and neutrophils) (89–91).

The molecular consequences of the PTPN22 mutation and the impact on T1D risk have been discussed extensively before (92, 93). The debate regarding the impact of the T1D risk variant on T cells remains as the results support both gain-of-function and loss-of-function as a mechanism (94). Following the gain-of-function hypothesis, the R620W mutation blunts the TCR signaling allowing autoreactive thymocytes to escape selection (95). The same group reported later that R620W is located in the P1 motif and prevents the binding of LYP to CSK (96), directing towards a loss-of-function hypothesis that may affect TCR signaling and certainly applies for the regulation of LFA-1 signaling. A deletion of *Ptpn22* in mice, mimicking the loss-of-function, demonstrated increased Treg levels (97) which is in line with a study that shows a direct correlation between having the *PTPN22* R620W variant and elevated circulating Treg frequency in humans (98). Recently, Perry et al. showed a higher expression of *PTPN22* in Tregs than in conventional T cells (Tconv) at rest irrespective of the genotype, but a lower impact of the risk variant on the suppression of distal TCR signaling in both subsets and permitting a stronger proliferation of Tconvs. The consequences for Tregs in this study were less evident implying a differential contribution of *PTPN22* risk variant to Tconv and Treg (99).

In myeloid cells, *PTPN22* is involved in the downstream signaling of TLR4, TLR7/8, NOD2 and cytokine receptors (reviewed in (92)). In this case, LYP does not work as a phosphatase but promotes TRAF3 ubiquitination and TLR-induced upregulation of type I interferons (IFNs). The *PTPN22* R620W variant failed to support this type I IFN response (100). Additionally, antigen presenting cells with the *PTPN22* R620W variant are more sensitive to NLRP3 and secrete more IL-1b in response to TLR-stimulation (101). Combined with the dampened type I IFN signal, this could affect the response of myeloid cells to infections (102) and the subsequent activation of T cells.

## PTPN2

Protein Tyrosine Phosphatase, Non-Receptor Type 2 (*PTPN2*) is ubiquitously expressed, including β-cells and hematopoietic cells. PTPN2 takes part in a broad range of signaling pathways regulating the response to hormones, cytokines and inflammation (93, 103–105). The β-cells upregulate PTPN2 in response to cytokines or polyI:C (mimicking viral infection) (106, 107). Judging by the effects of knockdown in primary rats and human β-cells, which exacerbated cytokine induced pro-apoptotic signaling *via* STAT1, JNK1, and BIM and enhanced apoptosis, PTPN2 plays a protective and anti-apoptotic role in β-cells (106–108). The risk SNP rs1893217 (OR=1.210) is an intronic non-coding variant which may contribute to the sensitivity of β-cells to immune- or virus-mediated apoptosis (107).

The risk variant of *PTPN2* is associated with decreased PTPN2 expression in CD4+ memory T cells and reduced IL-2 receptor signaling *via* STAT5 phosphorylation, which correlated with reduced FOXP3 expression in Tregs (109) suggesting that PTPN2 indirectly modulates IL-2 responsiveness in T cells and thus can work independent of the susceptible *IL2RA* gene variant. This dysregulation of Tregs can contribute to the faulty maintenance of autoreactive T cells and B cells and thus sustain the vicious circle of uncontrolled autoimmune response (93). Indeed, antigen-specific effector T cells (Th1 and Tfh) in *Ptpn2* deficient mice show increased proliferation (110). Cell cultures of human myeloid cells showed that a loss of *PTPN2* enhances IFN-g, IL-6 and MCP-1 secretion (103), implicating PTPN2 in the regulation of inflammation through antigen presenting cells as well.

## IFIH1

Interferon Induced with Helicase C Domain 1 (*IFIH1*) encodes for melanoma differentiation-associated gene 5 (MDA5). MDA5 is a cytoplasmic receptor for double stranded RNA (dsRNA) and detects viral RNA (106, 111, 112). Detection of dsRNA will activate a cascade of antiviral responses in the innate immune system by the production of IFN (113, 114). There are four SNPs in the *IFIH1* gene (rs1990760, OR=1.180; rs3747517, OR=1.700; rs2111485, OR=1.171; and rs13422767, OR=1.799) which are associated with T1D (70, 71). Variants rs2111485 and rs13422767 are located in an intergenic region of the 2q24 locus (13–23 kb 3′ of *IFIH1*), but it is not known whether the DNA sequences in this region act as a transcriptional silencer or enhancer. Winkler et al. showed that children at risk and islet-autoantibody positive with the rs2111485 variant genotype progressed faster to T1D (115). The contribution of other SNPs in the disease progression was not validated in this study. Variants rs1990760 and rs3747517 are located within the binding site of transcription factors and could therefore influence the expression of *IFIH1* (116).

Human PBMCs and cell lines with the *IFIH1* rs1990760 variant (coding an amino acid substitution A946T) have heightened basal and ligand-triggered IFN-I production (117). This SNP was thus characterized as a gain-of-function variant with a capacity to protect the carriers against specific viral challenges while promoting the risk for autoimmune diseases. This confirmed a hypothesis based on the results from previous studies in healthy individuals carrying the rs1990760 variant and animal models (118, 119), that this variant enlarges the risk for autoimmune disease by increasing the basal activity of IFN-stimulated genes through the recognition of self-dsRNAs without the need for a concomitant viral challenge.

MDA5 activation in DCs mediates cell maturation, increasing antigen processing and presentation through the expression of MHC class I chemokine receptors and co-stimulatory molecules (120), thus promoting the activation and expansion of inflammatory T cells (119). Hence, a heightened MDA5 activation can support the induction of autoimmunity *via* agitated DCs presenting the islet antigens to T cells in a pro-inflammatory rather than an anti-inflammatory context.

Next to the viral or cytoplasmic dsRNA, mitochondrial dsRNA released after β-cell stress could trigger the production of proinflammatory cytokines in individuals carrying the *IFIH1* risk variants (70, 121). Namely, the normal processing of the transcribed mitochondrial genome increases under stress causing a leakage of the mitochondrial dsRNA remnants into the cytosol (121, 122), where MDA5 recognizes these as damage-associated molecular patterns (DAMP). Hence, metabolic stress in β-cells that causes mitochondrial dysfunction might also contribute to the heightened IFN response and apoptosis of β-cells (123).

## CTLA4

The Cytotoxic T-Lymphocyte Associated Protein 4 (*CTLA4*) genes encodes a transmembrane co-receptor expressed on the surface of T cells. CTLA-4 functions as a negative regulator of T cell activation which can mediate T cell regulation or apoptosis by interacting with B7, a co-stimulatory molecule present on antigen presenting cells (124–127).

Genetic studies on *CTLA4* in T1D have been focusing on three gene variants: the A49G SNP (rs231775, OR=2.000) in exon 1, the SNP rs3087243 (OR=0.840) which is in high linkage disequilibrium with the dinucleotide (ATn) repeat in the 3’- untranslated regions (UTR) and the coding C318T SNP (rs5742909, OR=1.500) in the *CTLA4* promotor (128).

The first SNP rs231775 is in exon 1 at position 49 from A to G (A49G) of the *CTLA4* gene (129, 130). Meta-analysis of 76 studies showed that the rs231775 variant is more prevalent in T1D patients with Caucasian and South Asian origin and is associated with Type 2 Diabetes (T2D) in East Asians and South Asians (75). The A49G SNP causes the amino acid replacement of threonine to alanine and influences the posttranslational modification of CTLA-4. These modifications result in an inefficient CTLA-4 glycosylation and decreased expression of CTLA-4 on T cells, leading to uncontrolled T cell activation, including the autoreactive T cells (75, 131). The rs231775 variant was also associated with reduced production of soluble CTLA-4 (sCTLA-4), which can inhibit T cell proliferation by binding/blocking B7 (132). This has been confirmed in *Ctla4* KO NOD mice (133), in which the posttranscriptional silencing of sCTLA-4 reduced Treg potency and accelerated T1D onset. Interestingly, sCTLA4 suppressed proliferation of committed islet autoreactive T cell clones isolated from the blood of T1D patients in a dose-dependent manner, but it was unable to suppress naïve alloreactive T cells in an MLR (134), indicating a differential role for sCTLA4 in the control of memory versus primary immune responses.

The second SNP rs3087243 affects the size of dinucleotide (AT)n repeats in the 3’-UTR and the *CTLA4* mRNA stability through a post-transcriptional control (135), influencing the rate of translation (38, 136, 137). De Jong et al. showed that autoreactive T cells with long variants of the (AT)n repeat in the 3’-UTR region have reduced *CTLA4* mRNA levels (138), thus variations in the length of (AT)n repeats influence *CTLA4* expression contributing to the development of T1D. Also a rare genetic variation (rs13384548) within the 3’-UTR of the *CTLA4* mRNA disrupted the miR-302a* binding site reducing the capacity to control *CTLA4* mRNA (139).

The SNP rs5742909 in the *CTLA4* promotor region cause a C to T mutation at position 318. Individuals carrying the minor 318T allele have a higher promotor activity than the 318C allele, resulting in an increased expression of CTLA-4 by T cells (140). While this suggests that the C to T transition increases a regulatory function, the consequences for the T cell response and the effect of this gene variant on the development of T1D is not clear yet.

## IL2RA

The protein IL-2Rα (CD25) is a high-affinity subunit of the IL-2 receptor that forms a complex with IL-2Rβ- and γ-chain to activate intracellular signaling upon interaction with IL-2 (141). IL-2RA is constitutively expressed on Tregs and can be induced upon activation in other (effector) T cells (142). Polymorphisms in the genes encoding for the IL-2 receptor, *IL2RA* (rs2104286, rs61839660, rs10795791, and rs41295121) and *IL2RB* (rs743777), are associated with T1D (69, 76, 143, 144). DNA methylation at CpGs (−373 and −456) within the promotor of the *IL2RA* gene was slightly higher in T1D patients than in controls (142), indicating that epigenetic changes in the *IL2RA* promotor might participate in the *IL2RA* risk allele for T1D. Indeed, methylation at CpG-373 was correlated with 16 SNPs in the IL2RA gene, both with the protective alleles (rs12722495, rs2104286, rs61839660) and the susceptible allele at rs11594656 (**Table 1**) (11, 69, 145, 146).

Regarding the functional consequences for T cells, Dendrou et al. showed that individuals with the SNP rs12722495 (OR=0.620) had a higher CD25 expression on CD4+ memory T cells, while the carriers of the SNP rs2104286 (OR=0.880) showed a lower CD25 expression on naïve CD4+ T cells, compared to the non-carriers (147). They further demonstrated that individuals with the protective variant (rs12722495) consistently had higher proportion of activated IL-2 producing CD69^+^ CD4^+^ memory T cells compared to individuals with a susceptible allele, supporting the hypothesis that cells with a higher surface CD25 are more responsive to IL-2R mediated activation (147). This is consistent with the earlier observed defects in IL-2 production in T1D patients (148, 149). Cerosaletti et al. challenged the view that expression levels of CD25 functionally contribute to the susceptibility and showed a reduced signaling from IL-2R (measured by a phosphorylation of STAT5) in CD4+ CD25hi T cells of T1D patients and healthy individuals carrying the rs2104286 risk haplotype (150). The unexpected higher expression of CD25 on naive Tregs in T1D patients and healthy controls with the rs2104286 risk haplotype compared to the carriers of the protective variant, was not explained in this study. The rs2104286 haplotype also correlated with increased soluble IL-2RA levels, suggesting that shedding of the IL-2RA may account for the reduced IL-2R signaling in these individuals. Alternative hypothesis explaining the protective effect of the SNP rs12722495 and the contribution of polymorphisms in IL-2R-pathway in general was through the effects on nTregs (151). Given their constitutive expression of CD25 and a strong sensitivity to IL-2, lower IL-2 signaling measured by the STAT5 phosphorylation reduces the IL-2 response, impacting the FOXP3 expression and thereby affecting the inhibitory function of Tregs (151, 152).

The SNP rs61839660 (OR=0.620) is located within the *IL2RA* gene and it is a non-coding causal SNP variant for T1D (11, 60). This SNP is co-inherited with a so-called group-A protective T1D haplotype that also includes the rs12722495 (153). Interestingly, a rare variation in the group-A haplotype causing the loss of the protective allele only at SNP rs61839660 was sufficient to counteract the high *IL2RA* mRNA and surface CD25 expression (153). The mechanistic studies revealed that the minor SNP variant reduces the *IL2RA* enhancer activity (154, 155), which is stimulation-responsive causing a delay in CD25 expression upon T cell activation, and that a deletion of this enhancer diverted the effective Treg polarization in mice (155).

Monocytes-derived and myeloid DCs express CD25 both as a surface-bound and soluble molecule when stimulated with prostaglandin E2 (PGE2) (156). Also, tumor-associated DCs co-express CD25 and the inhibitory molecule IDO (156). In our hands, tolDC express lower *IL2RA* mRNA and lack the surface-bound CD25 compared to mDCs (17). We did not measure whether tolDC also release less soluble CD25. Taken together, the surface-bound CD25 may enable mDCs to catch IL-2 and use it to stimulate T cells, while the soluble CD25 molecule could work to block IL-2 and help the regulation of T cell responses (157). The contribution of *IL2RA* risk variants to the DC function has not been investigated. As the effects described so far in T cells predominantly impact the downstream IL-2R signaling and DCs do not express other two proteins of the IL-2R complex, the functional contribution of genetic polymorphisms in *IL2RA* is more likely to show through the surface expression or production of soluble IL-2RA than to impact DC differentiation.

## CD226

CD226 or DNAX-accessory molecule-1 (DNAM-1) is a transmembrane receptor expressed on T cells, NK cells, NKT cells, platelets and a subset of B cells (69, 158), and aids their activation and differentiation through co-stimulation (159). The inhibitory counterpart of CD226 is T cell Immunoreceptor with Ig and ITIM domains (TIGIT), which is a negative regulator molecule expressed in Tregs and NK cells (160). TIGIT binds CD155 on DCs, driving them towards a tolerogenic phenotype. Disturbance of the TIGIT/CD226 axis could therefore contribute to the development of autoimmunity (161).

The SNP rs763361 (Gly307Ser, OR=1.120) in the *CD226* gene is associated with multiple autoimmune diseases, such as T1D, multiple sclerosis (MS), autoimmune thyroid disease, RA, SLE and systemic sclerosis (162). This SNP results in a missense mutation at position 307 (glycine to serine) and is located in two intracellular phosphorylation sites of the protein (residue 322 and 329) (159, 163). The SNP rs763361 may alter RNA splicing by disrupting splice site enhancers or silencers, resulting in an isoform of CD226 with altered function (69, 159, 163) and increased CD226 activity in T cells (164).

Indeed, Gaud et al. showed that *in vitro* anti-CD226 and anti-CD3 co-activation of human primary CD4+ T cells of individuals carrying the rs763361 risk variant induces enhanced p-ERK (164). The ERK pathway regulates T cell activation and differentiation. The rs763361 variant is associated with skewing to Th17 and Th17.1 cells after stimulation *in vitro* (164). Indeed, T1D patients carrying the rs763361 risk variant had greater frequency of GAD antibody and low C-peptide levels, reflecting a more aggressive disease pattern in a Brazilian population (165). Wallace et al. observed that the rs763361 risk variant correlated with reduced CD226 mRNA levels in monocytes and which could reduce cell activation and thus alter the interactions between monocytes and lymphocytes (166). When *Cd226* was deleted in NOD mice, this decreased disease incidence and insulitis as compared to WT mice (167), but the deletion also increased the number of CD8+ thymocytes and splenocytes. The CD226 deficient CD8+ T cells showed decreased reactivity to the β-cell specific antigen IGRP, from which Shapiro et al. concluded that CD226 plays a role in the development of T1D by modulating thymic selection and affecting activation of CD8+ T cells (167). The effect of the rs763361 risk variant has not been studied in human CD8+ T cells or Tregs. The majority of human Tregs highly express TIGIT, but a Treg subset co-expresses CD226 (168). These CD226+ Tregs were associated with reduced suppressive capacity. Hypothetically, the rs763361 variant, which increases CD226 activity in T cells, will increase the proportion of CD226+ Tregs and thereby reduce the overall suppressive capacity of Tregs. Studying further the expression and function of CD226 in humans is needed for a better understanding of whether the rs763361 risk variant contributes through T cell activation only or also by affecting the interaction between monocytes and lymphocytes.

## Tolerogenic Modulation of Dendritic Cells and the Impact on the Minor Risk Genes

Gene expression can be changed by genetic engineering or using bioactive small molecules, for which aim the specific targeting of the scarce autoreactive T cells seems difficult. The targeting through DCs seems more viable and allows also antigen-specific immune modulation (169). The active form of VitD3 functions as a transcription factor upon binding to the vitamin D receptor (VDR) (170), creating a complex that binds with retinoid-X receptor (RXR) to enable the attachment to vitamin D response elements (VDRE) (171, 172). The VDR complex has a large effect on more than 3000 target genes, which includes forty-seven transcription factors and thus leaving hardly any immune pathway unaffected by VitD3 (173). This natural immunomodulator influences the development and function of T cells, B cells and monocytes (172, 174, 175), and controls the ability of the immune system to dampen inflammation. In two independent studies we found that about a third of the transcripts encoded by non-HLA T1D risk genes were differentially expressed between inflammatory mDCs and VitD3-derived tolDCs (17, 18). Interestingly, only five of these genes were also reported as direct targets of VDR (170), leaving others to an indirect control by VDR-targeted transcription factors. Of the direct VitD3-targets, the expression of *ORMDL3*, *SH2B3*, *IKZF1*, *PTPN2* and *IFIH1* genes was lower in tolDC while RAC2 and PTPN22 were higher in tolDC than in the inflammatory mDCs (17, 18).

The protein encoded by ORMDL3 is an enzyme involved in sphingolipid synthesis and lipid metabolism without a clear function in the immune response but interestingly the T1D patients who were the carriers of the linked polymorphism (rs12150079) showed a lower intensity of autoreactive T cell responses in T1D (11, 60, 176). The *SH2B3* encodes LNK (lymphocyte adaptor protein) that takes parts in several signaling pathways controlling the hematopoiesis, cytokine and integrin signaling and cell migration (177). The functional consequences of the T1D risk variant (rs3184504) that causes a missense mutation remain speculative (11, 59, 146), one study using human cells that reports an augmented lymphocyte proliferation that correlates with the predisposing gene variant (178) Interestingly though, a recent study shows that the T1D risk-gene variant associates with a reduced mortality from sepsis in individuals with a European decent and suggest based on a mouse model that augmented phagocytosis and myelopoiesis may be underlying mechanisms (179). The gene *IKZF1* codes for the transcription factor Ikaros (180), and the associated SNPs (rs10277986, rs62447205) are protective for T1D (11). How these SNPs affect the expression or function of Ikaros has not been described. Ikaros is a regulator of dendritic cell differentiation and immune homeostasis, and *IKZF1* deficiency causes less inflammatory cytokines secrection by human monocytes (181), which is in line with the observed lower expression in our tolDCs. Finally, *RAC2* encodes a protein from a Rho family of GTPases involved in cytoskeletal reorganization (e.g. needed for phagocytosis) but the effect of the described SNP variant (rs229533) increasing the risk for T1D is still unknown (11).

## Can a Model Based on an Integrated View on the Genetic Risk Help Us Treat Patients?

In our view, the polymorphisms in immune genes as are discussed in this review can influence both immune activation and regulation through a change in gene expression or in function (**Figure 1**). The consequences may differ between the cell types depending on the expression level or an implicated cellular function of a given gene. Indeed, by changing the expression of a target protein, some risk variants cause different functional effects in conventional T cells, Tregs or β-cells, depending on the implicated cellular function (**Figure 1**). For most of the evaluated genes, both the non-risk variant and the risk variant show opposing functional consequences in conventional T cells compared to Tregs. Namely, the risk genes for which a non-risk variant supports immune regulation (*PTPN2*, *CTLA4* and *IL2RA*) are indeed activating for Tregs and work to suppress the effector T cells. The risk-variant SNPs of these genes change the function in the same manner irrespective of the cell type but the end result differs so the lower expression and signaling through PTPN2, CTLA-4 or IL-2RA will simultaneously impair the function of Tregs and release the tight control of the effector T cell. Similarly, the activation-promoting function of CD226 in effector T cells, enhanced by the risk-SNP, suppressed the inhibitory function of Tregs. Further, based on the regular function of LYP (PTPN22) to control the post-TCR signaling events, the activating contribution of the risk mutation in Teff is evident but the consequences for the human Tregs remain to be confirmed. The risk-SNP induced modulation of *PTPN2* and *IFIH1* will increase β-cell apoptosis, which increases the antigen release, and thus contribute to the development of T1D. In DCs the *PTPN22* risk variant fails to promote upregulation of type I IFN which might result in diminished human host-protecting responses when dealing with viral infections. The *PTPN2* risk variant may dysregulates the production of inflammatory cytokines and thus the maintenance of immune tolerance by DCs. The *IFIH1* risk variants causes an increased IFN response, stimulating antigen presentation, while the *IL2RA* risk variant may inhibit the capacity of DCs to suppress T cell proliferation and cytokine production. In summary, the functional consequences of the causal T1D-risk variants have been extensively investigated and seem to paint a clear picture regarding the individual contributions but it is difficult from this information to consider how the polymorphisms may cumulatively modify the cell interactions that promote the impaired β-cell specific immune responses in individuals with high T1D risk-scores.

At the moment, no therapies have been developed that target *PTPN22*, *PTPN2*, *IFIH1* and *CD226*. Therapies targeting *CTLA4* (CTLA-4-Ig, abatacept) has been proven as safe and efficient to inhibit naïve T cell activation and therefore this approach is more selective namely inhibiting only T cell responses as compared to general/broad immunosuppression. The safety of abatacept as a subcutaneous (SC) and intravenous (IV) therapy is evaluated in RA (182, 183) and T1D patients, reporting no therapy-specific adverse effects (184, 185). Abatacept treatment showed good efficacy in prevention complete loss of β-cell function in T1D patients as is shown by preservation of C-peptide levels and insulin sensitivity improvement (184, 186, 187).

To compensate for the impaired IL-2 signaling due to lower expression or dysfunction of the IL2R gene, specifically in Tregs, ultra-low-dose IL-2 therapy has been tested, thus avoiding potential toxic effects of systemic IL-2. A phase II study has already been completed to determine the optimal IL-2 dose to use as a treatment in T1D patients (188). Participants did not exhibit severe adverse effects, a minimal NK cell expansion was observed after IL-2 treatment and no detrimental changes in glucose metabolism were observed, guaranteeing the safe use of IL-2 (188). Also, there was a dose-dependent increase in Tregs in all patients, and the low dose of IL-2 upregulated CD25 and FOXP3 expression on Tregs while CD4+ T effector memory cells were unchanged (189). Even though the latter trial showed some interesting effects of IL-2 therapy, the insulin secretion was not measured so the clinical efficacy of this therapy (i.e. on β-cell preservations) could not be determined. A newer alternative to avoid the influence on effector T cells uses the IL-2 mutant proteins (190), which has advanced to the clinical testing in GvHD (ClinicalTrials.gov Identifier: NCT03422627), though it remains a systemic antigen-independent approach.

Even though these therapies targeting *CTLA4* and *IL2RA* seem promising, it remains to be seen whether they are beneficial for all patients or only those carrying the affected variant, and to what extent such therapies may influence the immune system and health in general given the generic and pleiotropic effects of IL-2. It is tempting to investigate whether therapies targeting specific pathways in which a risk gene of interest is involved or epigenetic targeting of a single of multiple risk gene variants will be more beneficial.

A treatment that simultaneously tackles multiple changes in the risk genes allowing a correction towards the non-risk variant function could potentially aid as curative intervention. The natural immunomodulator VitD3 seems a good candidate since it reduced the expression of *IKZF1*, *PTPN2*, *IL2RA*, *CD226* and *IFIH1* while increased *RAC2*, and *PTPN22* in tolDC as compared to mDC. Considering the effects of the discussed risk-SNPs in these genes, the modulating action of VitD3 could counteract the immune-activating effects of risk-mutations in *IL2RA*, *CD226*, *IFIH1* and *PTPN22* while supporting the protective effects of *IKZF1*.

The potential clinical benefit of the treatment with Vitamin D, has been recognized earlier. The initial trial with VitD3-modulated tolDC in T1D patients confirmed safety and the clinical benefit of the treatment remains to be tested (191). VitD3 modulates T cells (172, 175, 192, 193) and a trial testing the combined treatment of T1D patients with VitD3 and GAD antigen did not show significant change overall but a particular β-cell preservation in individuals with the HLA DR3-DQ2 haplotype (194). Trials testing vitamin D supplementation (195–200), showed some clinical benefit such as improving diabetes control (HbA1c or insulin dose), reducing complications (195, 196), some indications of β-cell protection or immune regulation (197, 198), none of the study monitored the clinical and immunological effect simultaneously. Finally, VitD3 can contribute to T1D prevention since early postnatal VitD3 administration seems to protect from T1D (199), even though reduced circulating VitD3 levels do not increase T1D risk (200).

## Conclusions and Perspectives

Understanding the role of genetic risk-variants in the T1D pathogenesis can have important implications for better understanding disease pathogenesis and heterogeneity, as well as the development of specific/selective disease intervention strategies. Models have been generated suggesting that different T1D risk-loci contribute to successive pathogenic checkpoints, which detection could allow timely and appropriate modulation of the autoimmunity and increase the chance for curative interventions. The mechanisms involved in the immunotherapy of cancer (201, 202), teach us about genetic variants that increase the risk for the development of autoimmune disease but positively impact the survival after cancer treatment (203). Hence, polygenic risk scores (204) not only help to predict disease but also to predict when a specific patient is more or less likely to respond to immunotherapy directed at the involved pathways.

The genetic risk score could allow early identification of individuals who will develop T1D allowing earlier curative interventions. Butty et al. studied the frequency of non-HLA risk alleles among individuals at risk of developing T1D (DTP-1 trial), of which about one-third progressed to the clinical disease (205). They concluded that immune risk gene variants more likely condition the initial development of autoimmunity, resulting in a detectable auto-Ab response, but less critically contribute to the events leading to disease onset (205). Hence immune modulation therapy makes more sense prior to the onset of autoimmunity, which will be possible when a prediction of T1D is improved. The recently reported improved cumulative risk score (T1D-GRS2) that includes 67 SNPs (all HLA-DQ haplotypes, non-DR-DQ loci within the HLA region and non-HLA loci) indeed enabled a sensitive discrimination of T1D from T2D and controls (13), but also improved the prediction of future T1D in infants. Still, around 10% of all infants would have to be monitored to capture 77% of future T1D cases. In this study it remains unclear whether successive application of the HLA-score, followed by a non-HLA score would have further improved the prediction sensitivity. Importantly, this GRS failed to predict T1D in patients with different ethnicities, underscoring the need to study all-inclusive cohorts (206).

Alternatively, fine mapping genetic studies of previously known autoimmune loci will also help to find relevant genetic variants with strong effect on the development of T1D (11, 60, 207). The availability of large human whole-genome sequencing data sets, also allows detecting rare SNPs with large effect size on complex traits (208, 209). Forgetta et al. recently discovered three novel risk gene variants in large human whole-genome sequencing data sets of T1D patients (67). Hence, studying the human whole-genome sequencing data might lead to the discovery of gene variants, which will give a better understanding of the genetics behind the development of T1D and possibly predict therapy responses.

Taken together, current literature only partially explains the functional implications of the risk-SNPs to the development of autoimmunity in T1D. The efficient in-depth analyses of the immune response that can detect and monitor low-frequent autoantigen-specific cells and a better understanding of immune tolerance are needed to investigate and understand the functional contributions of genetic polymorphisms in different cells of the immune system. The same polymorphism can have opposing functional consequences depending on the cell in which the linked gene is expressed. Gaining insight into how the human genetics impacts functional immunity is therefore important to allow discrimination of relevant and treatable targets and for selecting proper immunotherapy strategies with the most benefit for patients or individuals at risk of developing T1D.

## Author Contributions

All authors listed have made a substantial, direct, and intellectual contribution to the work and approved it for publication.

## Funding

CG is supported by the Stichting DON and Dutch Diabetes Research Foundation (grant number 2020.10.011). TN is supported by Innovative Medicine Initiative 2 Joint Undertaking under grant agreement No 115797 (INNODIA), which receives support from the European Union’s Horizon 2020 research and innovation programme and EFPIA, JDRF and The Leona M. and Harry B Helmsley Charitable Trust. JZ is supported by the Dutch Arthritis Foundation (grant number LLP-16) and BR is supported by the Wanek Family Project for Type 1 Diabetes.

## Conflict of Interest

The authors declare that the research was conducted in the absence of any commercial or financial relationships that could be construed as a potential conflict of interest.

## Publisher’s Note

All claims expressed in this article are solely those of the authors and do not necessarily represent those of their affiliated organizations, or those of the publisher, the editors and the reviewers. Any product that may be evaluated in this article, or claim that may be made by its manufacturer, is not guaranteed or endorsed by the publisher.
